# PROTOCOL: Evidence and gap map protocol: Understanding pathways between agriculture and nutrition: An evidence and gap map of tools, metrics and methods developed and applied in the last 10 years

**DOI:** 10.1002/cl2.1035

**Published:** 2019-09-08

**Authors:** Thalia M. Sparling, Howard White, Suneetha Kadiyala

**Affiliations:** ^1^ IMMANA London School of Hygiene and Tropical Medicine London UK; ^2^ The Campbell Collaboration New Delhi India; ^3^ London School of Hygiene and Tropical Medicine London UK

## 1 BACKGROUND

### 1.1 The problem, condition or issue

The global food price crises of 2007–2008 and 2010–2011 drew attention to the need for addressing the underlying determinants of malnutrition in low‐ and middle‐income countries (LMICs; Brinkman, de Pee, Sanogo, Subran, & Bloem, 2010; Webb, 2010). Specifically, as the primary source of food and income in LMICs, agriculture received renewed focus. Making agriculture work for nutrition—nutrition‐sensitive agriculture—has climbed the international development agenda (Ruel, Alderman, & Maternal and Child Nutrition Study Group, 2013). More recently, given the sharp increase in diet‐related chronic diseases underpinned by overweight and obesity in LMICs and the threats of climate change to diets, attention has expanded to leverage food systems to optimize nutrition, health and environmental outcomes (Johnston, Fanzo, & Cogill, 2014). Donors, researchers and implementers mobilized research agendas to invest in understanding how to strengthen agriculture and food systems to realize nutrition outcomes sustainably.

Progress in this field in the last decade included three key developments:

- Development of conceptual frameworks to aid the investigation of agriculture‐food system and nutrition linkages, highlighting multiple direct and indirect complex pathways (Global Panel, 2015; Hawkes, Turner, & Waage, 2012; Johnston et al., 2014; Kadiyala, Harris, Headey, Yosef, & Gillespie, 2014; Lock et al., 2010; Masters et al., 2018).
- Empirical examination of the linkages between agriculture‐food systems and nutrition and the key pathways mediating or modifying these relationships and systematic reviews (Arimond & Ruel, 2004; Girard, Self, McAuliffe, & Olude, 2012; Ruel, Quisumbing, & Balagamwala, 2018).
- Experimental studies and novel methodological approaches, with improved rigour in testing conventional (e.g., homestead food production) and novel intervention models (e.g., market‐based interventions for nutrition; use of participatory videos).

These efforts led to widespread recognition of inadequate tools, methods and metrics to study the direct, indirect and dynamic relationships between in agriculture‐food systems and nutrition outcomes. There have been several calls to accelerate the development of innovative tools, methods and metrics to underpin the development of a robust scientific evidence base needed to guide policy investments in agriculture‐food systems for improved nutrition and health. In response to this demand, several projects and programmes were launched specifically to develop new research methods, including the DFID‐funded Innovative Metrics and Methods for Agriculture and Nutrition Actions (IMMANA) programme (Innovative Methods and Metrics for Agriculture and Nutrition Actions [IMMANA], 2018).

Research undertaken under IMMANA and others (CGIAR, 2018; Global Dietary Database [GDD], 2014; International Dietary Data Expansion Project [INDDEX], 2018; Sustainable and Healthy Food Systems [SHEFs], 2018) have built on existing theoretical underpinnings and have helped to refine hypothesized pathways, illuminating additional aspects and dynamics between agriculture or food systems and nutrition outcomes, such as food environments, environmental factors and food safety. The Food and Agriculture Organization (FAO) of the United Nations (UN) adopted the High‐Level Panel of Experts (HPLE) definition of a food system: “all the elements (environment, people, inputs, processes, infrastructures, institutions, etc.) and activities that relate to the production, processing, distribution, preparation and consumption of food, and the output of these activities, including socio‐economic and environmental outcomes” (HLPE, 2017). Agriculture and health are part of the broader milieu of a food system. As such, there has been a proliferation of innovations in programme design and implementation, as well as in metrics and methods and their application. While the body of evidence on effectiveness of food systems to improve nutrition has been recently summarized (Ruel et al., 2018), the portfolio of new methods and metrics has not. It is now necessary to take stock of these developments and plan for the future to support the production of effective and relevant research.

### 1.2 Scope of the evidence and gap maps (EGM)

Recognition of the multiple pathways through which nutrition impact is achieved brought about new conceptual frameworks, and along with them, new thinking in how to measure the complexity and dynamism within these systems. The innovations that emerged range from new technology to new indices to the application of methods from other fields. New metrics and methods have been developed throughout the pathways (household production, decision‐making, income, etc.) linking agriculture and nutrition.

In a standard effectiveness map, the row headings are interventions, and the column headings outcomes. In this map, those thematic pathways or domains will be considered the “interventions” through which nutrition is improved. We consider our “outcome” to be tools, metrics or methodological innovations, which are the columns of this map. Some innovations have been widely adopted across settings, and others are still in development. Therefore, each example innovation will be mapped using the studies that pertain to the innovation. Innovations have taken place at every level of measurement (individual, household, national, etc.) and correspond to certain cross‐cutting themes. These additional aspects will be coded internally on the map.

As an example of technology application at an individual and household level, researchers have utilized accelerometers to measure calorie expenditure in new ways to address intra‐household food allocation and gender roles (Zanello, Srinivasan, & Nkegbe, 2017). At a community level, researchers have employed wearable cameras and GIS technology to map changing food environments in urban areas (Schrempft, van Jaarsveld, & Fisher, 2017). A methodological innovation at a sub‐national level has been to use Bayesian theory and decision‐analysis for making policy that affects nutrition (Yet et al., 2016). More thorough data collection and new indices to capture prices of nutritious foods in markets at the regional level have led to better estimates of cost of nutritious diets in Ghana (Masters et al., 2018). New innovation in this field also includes tools to conceptualize and operationalize food systems, including how to frame and measure cost‐effectiveness of complex interventions, which have a range of outcomes (Masters et al., 2018). In this EGM, the columns will be types of innovation or novel application.

The aim of the gap map is to articulate and summarize the innovation in tools, metrics and methods that have been created and applied to understand food systems and agriculture‐nutrition linkages in the last ten years. We have chosen the ten‐year period based on the focus on and funding for agriculture‐nutrition linkages that emerged following the global food price crisis in 2007–2008, as well as wanting to focus on new innovations, which, by definition, would no longer be novel if developed more than a decade ago. We also aim to highlight gaps and opportunities for future development.

### 1.3 Conceptual framework of the EGM

Although the intervention‐outcome framework is most common for maps on effectiveness studies, this framework will be organized differently. We will take an approach that considers tools, metrics and methods (types of innovation/application in the columns) for agriculture‐food systems‐nutrition research (thematic domain in the rows). The map will be organized around a combination of conceptual frameworks that include the definition of food systems offered by the HPLE report on nutrition (HLPE, 2017), predefined pathways to improved nutrition (Global Panel, 2015; Hawkes et al., 2012; Herforth, Nicolò, Veillerette, & Dufour, 2016; Kadiyala et al., 2014), as well as additional themes that have been identified as more research is being undertaken on this topic (Grace et al., 2018; Masters, 2016; SHEFs, 2018) (Appendix A).

These conceptual frameworks (illustrated in Appendix A) overlap a great deal. For instance, each highlights the role of on‐farm production as a means for direct consumption as well as a potential income source, both which influence food availability and diet quality, and thus contribute to nutrition outcomes. Frameworks 1 (Kadiyala et al., 2014), 2 (Hawkes et al., 2012) and 3 (Herforth et al., 2016) are very similar—in fact the most substantive difference is the visual organization of components. These frameworks include aspects of women's time, income and employment. The same three highlight interacting aspects of care, education or knowledge, as well as overall health as drivers of nutrition outcomes.

Each framework also has differences, both in how it is visualized and the content. Each represents “indirect” determinants of nutrition outcomes, such as the role of climate, the environment, policy, governance and culture, inequity, and so forth, but some are shown as an outside layer of influence, whereas some of these are considered within the central framework. For instance, Framework 4 shows the interactions between environment or sustainability aspects and food production, highlighting that human health should always be balanced against planetary health, since they are symbiotic in the long run (Tuomisto et al., 2017). Several highlight important domains that are not equally represented on the other. Frameworks 5 (Masters, 2016) and 6 (Global Panel, 2015) propose the most current thinking about markets and the economic role of nutrition. These support the idea that production will lead to consumption only where, when and for whom markets are missing. The Global Panel Metrics and Methods Framework (Framework 6) puts the food environment as the central milieu into which other dynamics feed, and diet diversity, adequacy and safety as general by‐products of that food environment. In contrast, Framework 2 (Hawkes et al., 2012) specifically articulates the subsequent layers of the food environment that progressively lead to nutritional status. We will use all frameworks generally to ensure that the EGM is comprehensive and that the domains within conceptual pathways in agriculture to nutrition literature are represented and categorized logically, while maintaining iterative methods of refining the domains based on search results.

### 1.4 Why it is important to develop the EGM?

Governments, non‐government donors, implementing agencies and academia have all made significant investments, both intellectually and financially, in improving agriculture or food systems for nutrition outcomes. This investment has gone beyond scholarship and documentation and taken the form of application and innovation of tools, metrics and methods at every level. Stakeholders have called for a synthesis project on this topic in order to visualize the current portfolio of these developments, strategically plan the next wave of investment and shape the next generation of agriculture‐nutrition research.

### 1.5 Existing EGMs and/or relevant systematic reviews

There are no current gap maps on the topic of metrics and methods on the topic of agriculture and food systems for nutrition (or to improve nutrition outcomes). Some mapping exercises have been undertaken on pathways between agriculture and nutrition, namely the 2012 LCIRAH “Current and planned research on agriculture for improved nutrition: a mapping and a gap analysis”, which led the way to the IMMANA programme (IMMANA, 2018). The FAO Compendium of nutrition‐sensitive indicators also summarizes the most well‐established indicators on the subject (Herforth et al., 2016), but does not fully capture innovative tools and methodologies, as well as metrics that are in development currently. Furthermore, to our knowledge, none of these synthesis projects have been systematic or published as a formal EGM, and overall there have been no EGM of tools, metrics, or methodologies; rather most existing gap maps focus on effectiveness studies.

## 2 OBJECTIVES

The main objective of this EGM is to guide funders and researchers in the most promising areas of innovation within the study of food systems or agriculture to nutrition pathways, and demonstrate their phase of development and other thematic trends. We also will be able to demonstrate where there are gaps in existing innovative tools, metrics and methods that correspond to key domains identified in these conceptual frameworks. Empty cells in the map will indicate where no new methods, metrics or tools either exist or have been developed in the last decade within those domains. Furthermore, we intend that this EGM will then be used to shape future investments in this field, both by pursuing opportunities to take the most promising developments to the next level, and focusing attention on where there are gaps in available tools, metrics and methods. A secondary objective of this EGM is to identify trends in investigation and application that would be suitable for further synthesis.

## 3 METHODOLOGY

### 3.1 Defining EGM

All previous EGMs published to date have been compilations of effectiveness studies, therefore this EGM will be novel in many aspects. We are not aware of any protocols or published EGMs on which to model this project. There are several synthesis reports on this topic (Hawkes et al., 2012; Herforth et al., 2016), but as mentioned previously, none of them are current, systematic or are formal EGMs. We will use published, well‐established conceptual frameworks to define thematic domains of agriculture‐to‐nutrition in order to categorize the identified tools, metrics and methods. Therefore, our “intervention” will be each broad domain on the food systems or agriculture to nutrition pathway. The columns of our map, (outcome in effectiveness maps) will be each item of innovation (tool, metric or method) that has been developed or applied to capture or measure these domains.

While “innovation” is hard to categorically define, we will take a pragmatic, data‐driven approach to selecting what is new, novel or innovative in this dynamic field. By a data‐driven approach, we mean that we will use existing knowledge and data to inform the process, both through the published record and expert consultation. We will adopt several strategies and guidelines in selecting new, novel or innovative tools, metrics or methods:

● Limit the search to work published after 2008.
● Identify completely new tools, metrics or methods that were introduced after 2008 with no previous iterations.
● Identify tools, metrics or methods that existed prior to 2008 but have been significantly revised or modified since. As a “significant” change or modification is difficult to determine, we will rely on the group or authors' own assertions and explanations, and make an expert judgement as a group when unclear.
● Identify new, novel or innovative applications of existing tools and methods. This will mostly entail applying these cross‐disciplinarily. This will be the most difficult aspect of “newness” to define, and we therefore will also rely on the authors' description and justification, and secondarily make a collective expert decision.

Because some of the tools, metrics and methods are in their infancy, while others are globally adopted and have become standard practice, we will code each study based on the current (e.g. in 2019) stage of development of the innovation. We will further code and categorize innovations by several thematic filters (e.g., gender, equity, economics, technology, private‐sector engagement, conflict or political fragility), geographical application, and level of measurement.

### 3.2 EGM framework

The “intervention” (rows), will be defined using agriculture‐to‐nutrition conceptual frameworks mentioned previously, divided into “domains” of influence on agriculture and food systems or nutrition, such as household or on‐farm production, food policy and governance, or food environments and markets. The columns, or categories of innovations/applications, will be grouped by different classifications of tools (technology application and instruments to capture data on a range of agriculture‐nutrition topics), metrics (new indices and measures to quantify agriculture‐nutrition linkages) and methods (research design and analytical approaches applied to agriculture and nutrition research). We will code each study related to an item and group the items iteratively once all items have been mapped. We have chosen to do this since some well‐adopted innovations will have many papers that use the tool, metric or method, while others will have only a few, and some will apply the tool, metric or method in different ways. Users will be able to see each individual item as well as grouping by tool, metric or method.

Traditional EGMs include a quality assessment of each item, such as a risk of bias rating, which are not designed to evaluate tools, methods or metrics. In place of a quality assessment, this EGM will summarize the stage of development or application (explained below). We will add filter codes for certain cross‐cutting themes such as gender or private‐sector engagement. It will also categorize the measurement level (individual, household, district, national, etc.), and setting or geographical application (Asia, Africa, global, etc.). We also may add other filters as the search progresses. The framework structure is shown in Appendix B.

### 3.3 Population

This map will only include tools, metrics and methods that have been applied to agriculture‐nutrition pathways in any country at any level: individual, household, community or district, sub‐national, national and global.

### 3.4 Intervention

The problem we are considering is any domain that exists on the conceptual pathway between agriculture and/or food systems and nutrition outcomes. These domains have been grouped (through using frameworks and extensive rounds of expert consultation) by broad themes around food production, food safety, value‐chains, markets and food environments, food policy and governance, environment and climate, among others. We have organized the domains around broad themes in order to group items with minimal double‐ or triple‐coding, but we do envision that some items will appear in more than one domain. Whenever possible we will select a “primary” domain and use the filters and codes to indicate other aspects of the tools that are cross‐cutting, such as gender, technology or economics.

The first column lists 12 broad domains, and the second column are examples of what types of work would fit in to these domains. All included tools, metrics or methods must explicitly relate to either agriculture/food systems, or to nutrition. Any tools, metrics and methods that are not related to either agriculture/food systems or nutrition will be excluded. Most of the domains could be measured at various levels (individual, national, global, etc.). We will not differentiate in the domains, but rather in the internal coding of each tool, metric or method innovation. As the initial results are identified, we may refine these domains and add sub‐domains using an iterative methodology.

Specifically, we propose the following overarching categories based on the conceptual frameworks discussed previously (Table 1).

**Table 1:** Domains of influence on the agriculture or food systems to nutrition pathway

Domain	Examples (for illustrative purposes only—not exhaustive)
Primary food production (growing, cultivating, raising, catching, harvesting, storing)	Agriculture, agroforestry, aquaculture, husbandry as a source of food; on‐farm crop or food loss; yields; practices and techniques; harvesting; storage; processing for later consumption; seasonality; nutrient density/composition of crops; anti‐nutrients at the production level
Value chains and food transformation	Food processing for retail; food processing for storage and later consumption; retail food distribution; nutrient additions or losses or preservation (nutrition‐sensitive value chains); palatability; anti‐nutrients (or absence/removal) at the food transformation level
Food safety^a^	Aflatoxins; contamination; slaughter houses; wet‐market sanitation; food‐borne disease; bulking steps; food preparation in households and other sites
Water, sanitation and hygiene	Household water supply and water safety; distance to water; hygiene metrics; sanitation facilities; WASH checklists
Markets	Sale at markets; density; types; distance; accessibility; supply levels and availability; imports/exports; loss at market level
Economy	Purchasing power; consumption and expenditure; debt; economic resilience; income
Food environments^a^	Food quality; food diversity, food availability, food accessibility (prices, distance to stores), determinants of food access/value, i.e., any work that falls under the definition provided by the Centers for Disease Control: “The physical presence of **food** that affects a person's diet; a person's proximity to **food** store locations; the distribution of **food** stores, **food** service, and any physical entity by which **food** may be obtained; or a connected system that allows access to **food**”
	(https://www.cdc.gov/healthyplaces/healthtopics/healthyfood/general.htm.) Food environments can also be defined by the Nutrition Environment Measures Survey (NEMS) as: “Nutrition environments are places in the community where people buy or eat Food” (https://www.med.upenn.edu/nems/)
Ecology, sustainability and environment	Soil; forests; sustainability; climate change; resilience; water systems, agricultural water supply; water equity; biodiversity; land use
Policy and food governance, trade policy and commitments to nutrition	Commitments to nutrition (private/industrial/government); food prices; systems research and development; structural investments; trade regulation; tariffs, taxes, incentives (i.e., subsidies); institutional capacity, function and arrangements; decision‐making processes
Conflict of interest	Conflicts of food corporations; conflicting investments; manufacturing or supply of nutritious or unhealthy foods and marketing practices
Food insecurity	Food insecurity experience scales, methods for measuring seasonal food access and availability
Diet, nutrition and health	Nutrition KAP, norms and behaviours, food consumption, nutritional status indicators (e.g., energy balance, micronutrient status, anthropometry); NCDs; food production‐related labour burden, nutrition‐related child illness; diet quality; bioavailability

^a^ These domains have existing reviews that summarize the topic.

The domains of food safety, food environments and economic evaluations each have systematic reviews on methods and metrics. We will include the items that are included in those reviews and also use these items to test the framework.

### 3.5 Outcomes

The primary “outcome”, of the gap map (i.e., the columns in the map) is the innovative item (tool, metric or method) created and applied to studying and describing the broad agriculture to nutrition pathways. We will define “tool” as is a vehicle or an aid to collect information and data (e.g., a survey module to collect data required to compute an index or a piece of technology). “Metrics” will be defined as the parameters (measures) or indices used for measurement, comparison or tracking performance (e.g., disability adjusted life years; household dietary diversity score and Women's Empowerment in Agriculture Index [WEAI]). We define “method” as the process and approach involved in a systematic inquiry of relationships between agriculture, nutrition and health and generally refer to study design or application of an analytical method to this topic (e.g., impact evaluations using various types of counterfactuals, pathway analyses, decision analyses).

Several types of “innovation” are described above. We will identify each tool metric or method and group them together once identified. Some methods or tools will have slight variation in their application or analysis, but we will group these logically together as an item if the construction of the item is similar. We will include all of the studies on the map.

Each item might have multiple innovative components that fit distinctly within the categories of tools, metrics or methods. We propose that these will not be exclusive, rather the appropriate component of a paper or project will be categorized accordingly, and the total number of items summarized separately. In order to determine what is substantial enough for inclusion as a separate item (rather than a much smaller exploratory innovation and application), we will use the published or unpublished study objectives, considering only primary and secondary objectives. We will group tools, metrics and methods into the following categories, with some illustrative examples in the right‐hand column (Table 2).

**Table 2:** Categories of tools, metrics or methods used to study the agriculture or food systems to nutrition pathway

Category	Examples (for illustrative purposes only—not exhaustive)
**Tools (instruments for collecting data)**	
Technology measures/application	Instruments or other measurement tools: e.g., accelerometers, biological, physiological, testing mechanisms aflatoxin measurement techniques
	Geospatial applications: e.g., GIS, drones, spatial mapping
	Visual aids: e.g., wearable cameras, Photovoice
	Mobile/tablet‐based applications: e.g., mobile data collection
Survey and interview tools	Quantitative tools: e.g., Survey tools, new modules, new types of questionnaires
	Qualitative tools: e.g., new modules, new formats, new interview aids, new types of ethnography, focus groups, market surveys
**Metrics (measurements that correspond to outcomes of interest)**	
Measures: continuous, including scales	New Z‐scores
	New types or versions of Likert scales
	Assays, lab tests, vitamin A assessment
Indices: dichotomous or polytomous	WDDS, HDDS, MAHPF
	New classifications of growth measures, new body composition indices
**Methods (Organization or processing of scientific data)**	
Research design	Participatory design, surveillance systems, quasi‐experimental methods, diagnosis and validation
Analysis	Decision‐analysis; Bayesian theory; economic/cost analysis; optimization modelling; life tables; modelling studies

### 3.6 Criteria for including and excluding studies

#### 3.6.1 Inclusion criteria

● Must describe a tool, metric or method developed or applied (see definition in Section 3.5).
● Developed or updated since January 1, 2008.
● In grey literature, published and/or peer‐reviewed sources, as well as known projects from grant databases that describe tools, metrics and methods in development.
● In any country.
● Developed for the purpose of and/or used to quantify or qualify a potential interaction between agriculture or food systems and nutrition. This will be defined by the tool, metric or method being related to either the broader agriculture field, food systems as defined in the High‐level Panel of Experts (HPLE) Report or the broader nutrition field, since several methods and metrics used to study this interaction are only explicitly linked to one side of the pathway.
  ○ Nutrition or nutritional proxies on the outcome side of the pathway will be considered, including all forms of malnutrition (including diet‐related chronic conditions). Different kinds of nutrition measurement (food insecurity, diets, anthropometry, biomarkers, micronutrients), or diet‐related non‐communicable diseases (namely obesity, diabetes, CVD, or diet‐related cancers) will be included.
  ○ Examples: agricultural interventions to improve nutrition and their evaluation; assessing pathways to impact, the influence of agricultural practices and food value chains on nutrition; governance and policy processes through which agriculture and nutrition are linked; and links between climate, agricultural productivity and/or growth and nutrition at a macro scale, and so forth.
● Topics that have been linked to agriculture, food systems and nutrition pathways, as long as explicitly framed in relationship to these pathways and meet the eligibility criteria as new, novel or innovative. For instance:
  ○ Water insecurity and water footprints
  ○ Hygiene and sanitation, or water access
  ○ Livelihoods
  ○ Gender
  ○ Health care and care seeking
  ○ Trade/economics/markets

#### 3.6.2 Exclusion criteria

- Tools, metrics or methods not applied to the domains that link agriculture, food systems and nutrition, as explicitly defined by the HPLE or the conceptual frameworks included in the appendix.
- Tools, metrics or methods developed or applied prior to 2008.
- Projects with no reporting in English.
- In‐vivo laboratory animal studies. If the subjects are animals for agricultural production, livelihoods or consumption, they will be considered. If the animals are subjects as a proxy for humans or models for general interests (i.e., if the animals aren't to sell or eat), they will be excluded.
- In‐vivo plant studies not explicitly related to agricultural production, land use, or other related themes. Like the exclusion for animal studies, if the plants are specifically mentioned in the context of agriculture or consumption, even if lab studies, they will be included. If the plants are a model of general cell function or not mentioned in relationship to agriculture/ecology nexus, they will be excluded.
- Therapeutic nutrition.
- Enhancement nutrition.
- If the paper is related to nutrition and nutritional proxies, food supplementation for communicable diseases (e.g., TB, HIV), special groups or niche populations (such as hospital patients, athletes, etc.) will all be excluded.

#### 3.6.3 Types of study designs

The gap map will include primary research of any design, as certain study designs may in fact be an innovative method of application to study the agriculture‐nutrition linkages. Reviews will be excluded. Study types that may demonstrate new innovations or novel applications could include (a) a new study design, (b) standard study designs using new or innovative tools, metrics or methods, or (c) studies specifically developing, piloting or validating a new tool, metric or method. These would therefore include, as examples, a primary study describing how the approach or design is unique methodologically, a validation study, a technical manual or user guide for a new metric, or a newly developed impact evaluation methodology or an impact evaluation using well‐established study designs but using new tools or metrics. We will use the four considerations described above to determine what is considered “innovative” or “new” (Table 3).

**Table 3:** Examples of eligible studies

Title	Type of innovation	Domain	Filters
Filling a dietary data gap? Validation of the adult male equivalent method of estimating individual nutrient intakes from household‐level data in Ethiopia and Bangladesh	Measure (Metrics)	Diets (Nutrition and health)	Innovation: Stage 3
			Level of measurement: individual and household
			Setting: global
Piloting the use of accelerometry devices to capture energy expenditure in agricultural and rural livelihoods: Protocols and findings from northern Ghana	Instrument (Tools)	Primary food production	Innovation: Stage 1
			Level of measurement: individual
			Setting: Africa
			Gender, equity, technology
Validation of an Adapted Version of the Nutrition Environment Measurement Tool for Stores (NEMS‐S) in an Urban Area of Brazil. See NEMS definition of food environment.	Instrument (Tools)	Food environments	Innovation: Stage 2
			Level of measurement: community
			Setting: South America
The Women's Empowerment in Agriculture Index	Index (Metrics)	Primary food production	Innovation: Stage 4
			Level of measurement: individual
			Setting: Global
			Gender, equity

#### 3.6.4 Treatment of qualitative research

We will include qualitative research in the EGM as long as it fits within the agriculture/food systems to nutrition framework and meets the inclusion and exclusion criteria. Projects could be undertaken as part of a mixed‐methods project, or be entirely qualitative. Traditional methods such as mini‐ethnography, focus group discussion, or individual interviews will have to be innovative or applied in new ways to be considered for this EGM. Qualitative documentation of the food environment through participatory GIS, using technologies such as PhotoVoice to elicit consumer preferences, or employing a new visualization technique within a survey are all examples of innovation in qualitative methods that will be included in the EGM.

#### 3.6.5 Types of settings

Any innovation or application of tools, metrics and methods taking place anywhere will be considered. They could be from a specific country or region, or they could be applied globally, with relevance to LMICs (analytical approaches, trade issues, etc.).

#### 3.6.6 Status of studies

We will include ongoing studies and projects, identified through expert consultation, interviews, grant databases and unpublished documentation.

#### 3.6.7 Search strategy and status of studies

We will employ a comprehensive literature, trial and project database search that includes electronic screening with search terms (listed in Appendix C), consultation with subject‐matter experts (outlined in Appendix D). We will also search various project and research databases and key websites and backward‐track citations in the bibliographies of key papers, both listed below.

The following databases will be searched electronically:

– CAB Abstracts.
– Web of Science (seven databases).

The following websites will be searched:

– CGIAR research library: IFPRI, Bioversity, World Agroforestry, and International Livestock Research Institute
– DfID Research for Development Outputs
– BLDS
– FAO AGRIS
– IMMANA grant database
– The 3ie impact evaluation database
– IPA and J‐PAL since 2015
– The World Bank IEG evaluations
– USAID Development Experience Clearinghouse

The following databases/journals will be hand‐searched:

– The proceedings of the Agriculture, Nutrition and Health Academy conference
– The proceedings of the CSAE Conference
– The proceedings of the NEUDC Conference
– The World Bank Economic Review
  The key publications we will use for backward‐citation tracking are:
– Girard et al. (2012)
– Global Panel (2015)
– Hawkes et al. (2012)
– Herforth et al. (2016)
– Kadiyala et al. (2014)
– Masset, Haddad, Cornelius, and Isaza‐Castro (2012)
– Ruel et al. (2013)
– Ruel et al. (2018)
– Webb (2013)
– Some included articles, studies and reports

#### 3.6.8 Screening and selection of studies

We will use a traditional method of two independent researchers to search and then screen (both on title/abstract and full text) the first 10% of items, with a third researcher (Howard White, Denny John or Thalia Sparling) providing a decision in the case of disagreement. For the remaining search results, we will use single screening with 5% randomly checked by a senior researcher. We do not plan to use any automation or text‐mining. The Campbell Collaboration, in collaboration with the IMMANA team, will hire and manage an experienced team to complete the initial search, screen titles and abstracts according to the inclusion and exclusion criteria, and further screen full‐text publications. We will then collaboratively extract the data, code the projects and complete the coding for stage of innovation or application, which will replace a traditional quality assessment in this EGM.

#### 3.6.9 Data extraction, coding and management

Besides the rows of “intervention” and columns of “outcomes” described previously, we propose to code for several other factors that will act as filters in the EGM. Some filters will have predefined categories:

- Stage of innovation or application (definition provided in Quality Appraisal):
  - Concept development and pilot
  - Feasibility, efficacy or internal validity
  - Demonstration and testing, effectiveness and external validity/construct validity
  - Adoption, generalizability and widespread application
- Measurement unit:
  ○ Individual
  ○ Household
  ○ Community/sub‐district
  ○ District/sub‐national
  ○ National
  ○ Regional
  ○ Global
- Setting or geographical application (Africa, Senegal, Asia‐Pacific, global, etc.)
  Other filters will be used to identify cross‐cutting themes, which will not have categories:
  ○ Private‐sector engagement
  ○ Economics (e.g., cost‐benefit, cost‐effectiveness, cost of diets)
  ○ Gender dynamics and parity (Could be about men or women—anything about gender dynamics, e.g., decision‐making power over productive resources; employment in agriculture; self‐care and health decision‐making; social gender cohesion; women's mobility; leadership)
  ○ Equity (e.g., caste/ethnicity, economic, geographic, age)
  ○ Technology
  ○ Political fragility/conflict
  ○ Diets

The filters without categories will be applied if the authors of the item specifically state the relationship of the item to that theme or the research item is clearly linked to a particular filter. The same research team in charge of the search and screening will complete the coding of these filters and categories, which will have been decided through consultation with subject experts and a piloting one grant portfolio, but may also be modified once search results are identified. We will screen and code articles using EPPI Reviewer 4.

### 3.7 Quality appraisal

Traditionally, effectiveness studies included would be evaluated for risk of bias or overall quality. These quality assessments are not applicable when evaluating a tool, metric or methodology. However, we recognize that some formal assessment and/or ranking of each item included (tool, method or metric) will be useful to readers. In this map, some tools, metrics and methods will be too new to be widely adopted and tested across settings. As they may still be in development, we will draw on the traditional epidemiological lens of indicator development as well as stages of innovation to create four categories of development. During indicator development, various aspects of validity and reliability are explored and tested, which are all equal components in creating a successful indicator (Frongillo, 1999). In the Stages of Innovation, there is a hierarchical progression through ideas, proof‐of‐concept, design and wider application (Imperial College Health Partners, 2018). The overarching components of each are described below (Table 4).

**Table 4:** Comparison of indicator development guides with stages of innovation

Indicator development	Stages of innovation (modified from http://pathwaytoinnovation.co.uk/innovation‐stages)
	0. Preliminary research: investigating the opportunity for your idea, researching need for it, the potential demand and market
(1) Its construction is well‐grounded in an understanding of the phenomenon	1. Basic Technology research: translation of research and thinking into applied research and development, technology concept and/or application is formulated and practical applications identified, proof‐of‐concept
(2) Its performance is consistent with that understanding	2. Feasibility and development: Component validation in laboratory environment and basic integration of components to achieve a suitable level of performance. Secondly, component validation in relevant environment, testing fidelity and real‐world utility of project or technological components
(3) It is precise within specified performance standards	3. Demonstration: Show prototype or model of innovation in a relevant environment. Incorporating feedback gives management or funders confidence and advances R&D requirements.
(4) It is dependable within specified performance standards	4. Testing: Innovation is completed and “flight qualified” through test and demonstration, trials allowing for any “bug fixing” aspects of system development
(5) It is accurate within specified performance standards	5. Adoption and spread: Proving an innovation works in real life and persuading users to adopt

By using both the principles of indicator development as well as the progressive stages of innovation, we propose four categories of development or application:

1. Concept development and pilot: there is well‐defined problem which leads to a need for the innovation and a pilot innovation developed, the innovation is well‐grounded in an understanding of the phenomenon.
2. Feasibility or internal validity: the innovation is feasible within a controlled setting and demonstrably can address the problem it intends to address in initial testing. Reliability has been demonstrated.
3. Demonstration and testing and external validity: the innovation captures what it intends to capture on a larger scale, across multiple settings or in less controlled environments
4. Adoption, generalizability and widespread application: the innovation can be applied across multiple settings and contexts, measures what it intends to measure and is adopted by multiple stakeholders.

Singular items that describe a new tool, metric or method as it is developed or piloted will be coded as Stage 1. Items that are presented in a content validation or similar manner will be coded as Stage 2. Items that show evidence based on relationships with external variables (criterion, convergent, or discriminant validity) or application across or to new settings will be coded as Stage 3. More than five items mentioning a tool, metric or method developed or applied in a novel way after 2008 will be grouped into a simple count and used as evidence of “widespread” application, or Stage 4 of innovation. In these cases, the first five key papers will be coded and included.

## 4 ANALYSIS AND PRESENTATION

### 4.1 Unit of analyses

The unit of analysis will be the item (tool, metric or method) that develops the innovation or application. There may be several components of innovation, which will populate the subsections of tools, metrics or methods. Therefore, the total number of studies or projects will be calculated separately from the items of innovation, as one project may be categorized as having an innovative tool that is distinct from an innovative metric, for example, and several studies may be associated with a single item. Items will only be included in this EGM if they state the innovative tool, metric or method as either their primary or secondary objective, which will exclude minor or exploratory components of projects.

### 4.2 Planned analyses

We will present the EGM with a numeric summary of included items, broken down by thematic domain (intervention) and innovation type (outcome), as well as other subgroups such as stage of development or unit of measurement. We will also present a narrative report synthesizing several aspects of the EGM, described below. In the case of “unsuccessful” methods, metrics and tools, which do not result in publications because of their flaws, these projects will be documented and included, but not analysed in depth. It would be useful to map these so as to learn from their shortcomings when we move to the evidence synthesis stage. We will attempt to identify these through expert consultation.

### 4.3 Presentation

In this EGM, the rows of “intervention” will be various domains of influence along the agriculture or food systems to nutrition pathways, subdivided into several thematic domains such as household or farm production, women's role in agriculture, or food and farm policy and governance. These domains are derived from the conceptual frameworks in Appendix A, and listed fully in Appendix B. The columns of the map will group items by tools, metrics and methods, which will be further categorized into tools: technology application and survey and interview tools; metrics: measures and indices; and methods: research design and analysis.

Furthermore, we will internally code for the following characteristics, which will be modified or added to iteratively:

– Stages of development (described in quality appraisal) in place of a traditional quality assessment:
  - Concept development and pilot
  - Feasibility, efficacy or internal validity
  - Demonstration and testing, effectiveness and external validity/construct validity
  - Adoption, generalizability and widespread application
– Measurement unit:
  - Individual
  - Household
  - Community/sub‐district
  - District/sub‐national
  - National
  - Regional
  - Global
– Setting or geographical application
  - Africa, Senegal, Asia‐Pacific, global, etc.

Cross‐cutting theme filters:

- Private‐sector engagement
- Economics (e.g., cost‐benefit, cost‐effectiveness, cost of diets, purchasing power)
- Gender dynamics and parity (Could be about men or women—anything about gender dynamics, e.g., decision‐making power over productive resources; employment in agriculture; self‐care and health decision‐making; social gender cohesion; women's mobility; leadership).
- Equity (e.g., caste/ethnicity, economic, geographic, age)
- Technology
- Political fragility, conflict
- Diets

We will use the intervention (domain between agriculture and nutrition) and the outcomes (innovation items), as well as these domains to create the tables and figures, which will be decided finally based on our findings. Preliminarily, we propose a section of the report summarizing interventions, and another to provide a narrative summary of the outcomes. The report will include a section discussing the overall stage of innovation assessed in the map. We will include a section that highlights gaps and under‐researched areas for future investment (but not prioritized as this is subjective based on the user), as well as compare the portfolio of innovation to the existing conceptual frameworks used. Tables and figures may include:

- A PRISMA diagram
- A visual representation of the trend in innovations
- Countries or regions with most application or innovation
- A visual and numeric summary of pathways with the most innovation or application

## 5 STAKEHOLDER ENGAGEMENT

This gap map will be undertaken in close collaboration with the core IMMANA management and steering committees. IMMANA management committee includes experts from the Leverhulme Center on Integrative Research on Agriculture and Health (LCIRAH), the London School of Hygiene and Tropical Medicine (LSHTM) and Friedman School of Nutrition Science and Policy at Tufts University. IMMANA steering committee includes representatives from DFID, the Bill and Melinda Gates Foundation, USAID and CGAIR's A4NH programme. The lead authors will first work with this team to develop the framework and protocol, as well as solicit grey literature and unpublished materials. After that, we will send the draft framework and protocol to a wider group of experts in the field of agriculture‐nutrition research, including experts from the University of South Carolina School of Public Health, Johns Hopkins University, the Food and Agricultural Organization of the United Nations (FAO), the International Food Policy and Research Institute (IFPRI), other CGIAR institutions and IMMANA Grantees among others.

## ROLES AND RESPONSIBILITIES

- Content: The methodological and thematic framework development will be led by Suneetha Kadiyala, a leading expert in nutrition‐sensitive agriculture and the Principle Investigator of the IMMANA programme, supported by Thalia Sparling, a postdoctoral research fellow also working on food‐based approaches to improving nutrition. Dr. Kadiyala is an assistant professor at the London School of Hygiene and Tropical Medicine (LSHTM) with decades of work focusing on nutrition. Dr. Sparling is an epidemiologist and nutrition researcher who has been working in the field of public health for over a decade.
- EGM methods: Howard White, CEO of the Campbell Collaboration and a veteran systematic review expert, will lead the EGM methodology and advise the content experts on framework and protocol, as well as helping train and coordinate the information retrieval.
- Information retrieval: Information retrieval will be done by experienced teams hired and managed by the Campbell Collaboration, in collaboration with Thalia Sparling and Suneetha Kadiyala.

## SOURCES OF SUPPORT

This work is funded through the IMMANA programme, which is supported by the U.K. Department for International Development (DFID). We aim to submit the gap map Title Registration Form by August 31, 2018, the Protocol by March 15, 2019 and the have a draft gap map to deliver to DFID by June 15, 2019.

## DECLARATIONS OF INTEREST

None.

## PRELIMINARY TIMEFRAME

Approximate date for submission of the EGM: June 15, 2019.

## PLANS FOR UPDATING THE EGM

We are currently raising funds for a follow‐on research programme to the first IMMANA programme. If IMMANA 2 is successfully funded, we will update the EGM at the conclusion of the project cycle, approximately at the end of 2024 or beginning of 2025.

## APPENDIX A CONCEPTUAL FRAMEWORKS OF AGRICULTURE/FOOD SYSTEMS PATHWAYS TO NUTRITION

FRAMEWORK 1:

Kadiyala et al. (2014):

FRAMEWORK 2:

Hawkes et al. (2012):

FRAMEWORK 3:

Herforth et al. (2016):

FRAMEWORK 4:

Tuomisto et al. (2017):

FRAMEWORK 5:

Masters (2016):

FRAMEWORK 6:

Global Panel (2015):

## APPENDIX C SAMPLE SEARCH TERMS

Database: CAB Abstracts <1990 to 2018 Week 48>

Search Strategy: December 13 2018

‐‐‐‐‐‐‐‐‐‐‐‐‐‐‐‐‐‐‐‐‐‐‐‐‐‐‐‐‐‐‐‐‐‐‐‐‐‐‐‐‐‐‐‐‐‐‐‐‐‐‐‐‐‐‐‐‐‐‐‐‐‐‐‐‐‐‐‐‐‐‐‐‐‐‐‐‐‐‐‐

1. analytical methods/ or analysis/ or statistical analysis/ or methodology/ or experimental design/ or monitoring/ or measurement/ or data collection/ or models/ or mathematical models/ or environmental assessment/ or evaluation/ or performance indexes/ or program evaluation/ or social impact/ or environmental impact/ or impact/ or health impact assessment/
2. econometric models/ or econometrics/ or economic analysis/ or economic evaluation/ or economic theory/ or economic impact/ or cost effectiveness analysis/ or economic impact/
3. ("metrology" or "methods").id.
4. (new or original or unconventional or experimental or inventive or modern or advance* or innovat* or novel or introduc* or inaugurat* or launch* or recent* or up‐to‐date or updated or "not previously available" or emerging or validat* or adopt*).ti,ab.
5. ((new or original or unconventional or experimental or inventive or modern or advance* or innovat* or novel or introduc* or inaugurat* or launch* or recent* or up‐to‐date or updated or "not previously available" or emerging or validat* or adopt*) adj1 (method* or metric* or econometr* or metrolog* or measurement* or indicator* or meter* or module* or analy* or technolog* or technique* or application or device or tool or tools or toolkit*)).ti,ab.
6. ((1 or 2 or 3) and 4) or 5
7. (method* or metric* or econometr* or metrolog* or measurement* or indicator* or meter* or module* or analy* or technolog* or technique* or application or device or tool or tools or toolkit*).ti,ab.
8. 1 or 2 or 3 or 7
9. exp agriculture/ or agricultural research/ or agronomy/ or farming/ or farming systems/ or exp horticulture/ or horticultural crops/ or market gardens/ or pastures/ or crop production/ or crop husbandry/ or crop losses/ or livestock/ or native livestock/ or animal husbandry/ or livestock farming/
10. (agriculture or agro‐forestry or agroforestry or farming or horticulture or livestock or aquaculture or "fish farming" or ((food* or crop*) adj2 (produc* or grow* or cultivat* or rais* or harvest* or loss* or stor*)) or husbandry).ti,ab.
11. foods/ or food production/ or food safety/ or food processing/ or food storage/ or food storage losses/ or food environment/ or food deserts/ or food consumption/ or food policy/ or food security/ or food legislation/ or food marketing/ or food prices/
12. (food* adj2 (produc* or safety or process* or loss* or stor* or policy or policies or security or insecurity or consum* or environment or legislat* or market* or price or prices)).ti,ab.
13. exp nutrition/ or diets/ or nutrition research/ or nutrition surveys/ or nutritional assessment/ or nutritional state/ or nutrition programmes/ or nutrition security/ or community nutrition/ or nutrition policy/ or preventive nutrition/
14. (nutrition* or diet* or malnutrition or malnourish* or undernourish*).ti,ab.
15. or/9–14
16. 6 and 15
17. limit 16 to yr="2008 ‐Current"
  Annotation: New+Metrics+Ag/Nut/Food
18. 8 and 15
19. limit 18 to yr="2008 ‐Current"
20. ("farm diversity score" or "functional diversity index" or ("household* food*" adj3 months) or ("women's empowerment" adj2 agriculture) or ("food loss*" adj2 "supply chain*") or "global food loss* index" or "foodborne disease* burden" or "food safety score" or (coliform* adj2 milk) or (chloramphenicol adj2 residue*) or (diarrh* adj3 (child* or infant*) adj2 (prevalen* or epidemiolog* or distribut*)) or (water adj2 (distance* or collect*)) or (access* adj2 water adj2 (clean or improved)) or (cost* adj3 (diet* or "nutrient adequacy")) or (sale* adj2 (agricultur* or farm*) adj product*) or "household economy analysis" or "coping strateg* index" or ("household food insecurity" adj2 "access scale") or "food insecurity experience scale" or "household hunger scale" or "food consumption score" or "nutrition environment measurement tool* for stores" or (access* adj2 "healthy food") or "modified retail food environment" or "vulnerability and capacity assessment" or "nutrition* indicators for biodiversity" or "water footprint*" or ("soil quality" adj2 indicator*) or ("local authorit*" adj2 (response* or responsive*)) or (inclusive* adj2 participat* adj2 budget*) or (multi‐stakeholder* adj2 partner*) or (conflict* adj2 interest adj2 safeguard*) or (access* adj2 "basic service*") or ("minimum dietary diversity" adj2 (women or child*)) or ("dietary diversity score" adj2 (women or household*)) or "minimum acceptable diet*" or "non‐staple food energy" or "Shannon diversity" or "modified functional attribute diversity" or "nutrient* density score" or (nutrient* adj2 (intake or diversity or adequacy or availability)) or "nutrition* diversity" or ((diversity or bioversity) adj2 gradient*) or ("population share" adj2 "adequate nutrient*")).ti,ab,sh.

## APPENDIX D PRELIMINARY LIST OF EXPERTS

1. Suneetha Kadiyala
2. Will Masters
3. Alan Dangour
4. Rosemary Green
5. Jeff Waage
6. Bhavani Shankar
7. Elaine Ferguson
8. Andy Jones
9. Ed Frongillo
10. Marie Ruel
11. Jess Fanzo
12. Amy Webb‐Girard
13. Sera Young
14. Delia Grace
15. David Sterling
16. IMMANA grantees
17. Inge Brauer
